# Eradication of Ebola Based on Dynamic Programming

**DOI:** 10.1155/2016/1580917

**Published:** 2016-05-25

**Authors:** Jia-Ming Zhu, Lu Wang, Jia-Bao Liu

**Affiliations:** ^1^School of Statistics and Applied Mathematics, Anhui University of Finance and Economics, Bengbu 233030, China; ^2^School of Finance, Anhui University of Finance and Economics, Bengbu 233030, China; ^3^School of Mathematics and Physics, Anhui Jianzhu University, Hefei, Anhui 230601, China

## Abstract

This paper mainly studies the eradication of the Ebola virus, proposing a scientific system, including three modules for the eradication of Ebola virus. Firstly, we build a basic model combined with nonlinear incidence rate and maximum treatment capacity. Secondly, we use the dynamic programming method and the Dijkstra Algorithm to set up M-S (storage) and several delivery locations in West Africa. Finally, we apply the previous results to calculate the total cost, production cost, storage cost, and shortage cost.

## 1. Introduction

The Ebola virus large outbreaks in Africa began in 2014. The high number of people infected and the high mortality caused widespread concern in the world. Ebola virus disease shocked the world in 1976. It turned up for the first time in two cases that began the epidemic at the same time [1]. Another one is in the Democratic Republic of the Congo, which occurred near the Ebola River in a village [2].

Now the spread of Ebola virus has caused wide public concern all over the world. In order to make drugs and vaccine exert the greatest effect which can effectively cure patients, we propose a scientific system, including three modules for the eradication of Ebola virus. First, we build a basic model combined with nonlinear incidence rate and maximum treatment capacity. Then, we use the dynamic programming method and the Dijkstra Algorithm to set up M-S (storage) and several delivery locations in West Africa. Finally, we apply the previous results to calculate the total cost, production cost, storage cost, and shortage cost.

We established a practical, sensitive, useful model. For other manuscripts, we find that iteration, Floyd algorithm, and genetic algorithm are used to optimize the eradication of Ebola. Meanwhile, in our model, we consider not only the disease propagation speed and the drugs required quantity and the impact of transportation on the treatment but also the design of a distribution optimization feasible transmission system [3]. In addition, transmission sites and vaccine and drug production speed are points we should consider in building the model. Finally, we can apply our model to completely eradicate Ebola or at least alleviate the current tense situation [4].

## 2. How to Restrain the Spread of Ebola

### 2.1. Epidemic Model

Considering the production and distribution of drugs and local medical infrastructure, we build a basic epidemic model with maximum treatment capacity. How to restrain the spread of Ebola virus is shown in the following five steps.

**Step 1 .**

Build the epidemic model with nonlinear incidence rate and maximum treatment capacity.

**Step 2 .**

The total population changes because of the birth rate and natural death rate and is classified into *S*(*t*), *E*(*t*), *I*(*t*), *Q*(*t*), and *R*(*t*).

**Step 3 .**

Build the improved SEIQT epidemic model.

**Step 4 .**

Utilize the equivalent system of equations to figure out the basic reproduction number *R*_0_. If *R*_0_ < 1, the epidemic gets controlled and has a disease-free equilibrium, while it continues to spread and has endemic equilibrium if *R*_0_ > 1.

**Step 5 .**

Analyze the stability.

#### 2.1.1. Model Preparation

In most of classical epidemic models, the incidence rate is assumed to be *λSI* (*λ* is contact rate) which is a bilinear function of *S* and *I*. However, in fact, many infectious diseases have periodic fluctuations that might be caused by some external factors such as age structure, seasonal variations, and time lag or resulted from nonlinear incidence rate. To fit the actual situation and simplify the model, it is proper to use nonlinear incidence rate. Liu used it in the form of *βS* ^*p*^ *I* ^*q*^  (*p*, *q* > 0) [5, 6]. In this paper, we set *p* = 1 and *q* = 2.

#### 2.1.2. Model Establishment

**Figure 1:**
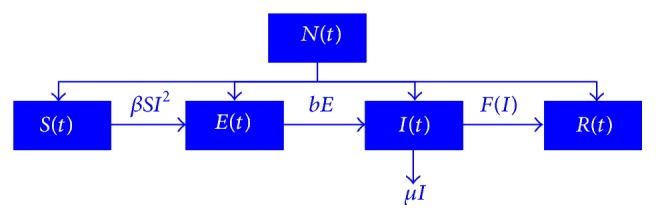
Overview of epidemic model.

We assume that there is no birth rate or natural death rate and the total population is fixed. The process of modeling is shown in Figure 1.

Build simultaneous differential equations:

(1) S′t=−βSI2,E′t=βSI2−bE,I′t=bE−μI−FI,R′t=FI,

associated with maximum treatment capacity:

(2) S′t=−βSI2,E′t=βSI2−bE,I′t=bE−μI−kI,R′t=kI,

where 0 ≤ *I* ≤ *I*_0_,

(3) S′t=−βSI2,E′t=βSI2−bE,I′t=bE−μI−kI0,R′t=kI0,

where *I* > *I*_0_.

From *S*(*t*) + *E*(*t*) + *I*(*t*) + *R*(*t*) = *N*(*t*), we can readily get *N*′(*t*) = −*μI*.

At the same time, the function *N*(*t*) is the total population of a region or country, the function *S*(*t*) is individuals who are susceptible to the disease, the function *E*(*t*) is individuals who are infected but without paroxysm, the function *I*(*t*) is individuals who are infectious, the function *Q*(*t*) is isolators, and the function *R*(*t*) is individuals who are removed. The function *F*(*I*) is individuals who are cured by the medication, the parameter *β* is proportion of the effective contact in the total population, and the parameter *b* is proportion of transformation from being infected to being infectious. The parameter *μ* is mortality due to illness of the infections, and the parameter *βSI* ^2^ is nonlinear incidence rate.

### 2.2. Improved SEIQR Epidemic Model

First of all, we must introduce the concept of basic reproductive rate. The basic reproduction number (sometimes called basic reproductive ratio and denoted by *R*_0_) of an infection can be thought of as the number of cases that one case generates on average over the course of its infectious period, in an otherwise uninfected population [7].

#### 2.2.1. Data Processing

To analyze the spread rate of Ebola, we collect the data of population and total cases and deaths in Guinea, Liberia, and Sierra Leone from May 27 to November 28 in 2014; see Table 1.

**Figure 2:**
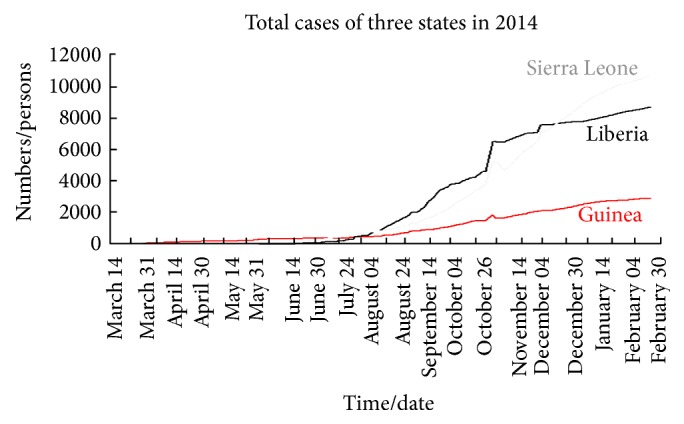
Total cases of three states in 2014.

**Figure 3:**
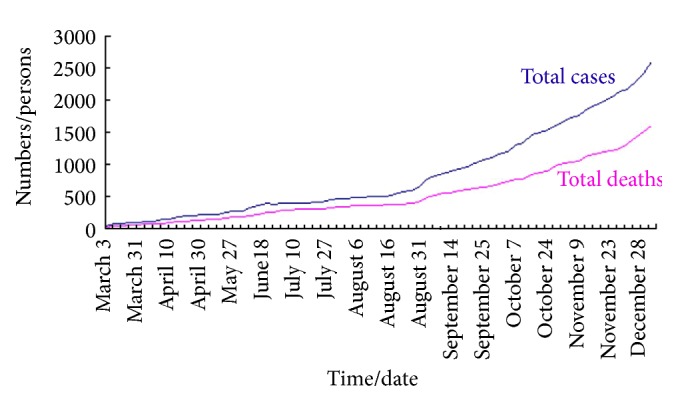
Total cases and deaths in Guinea.

**Figure 4 fig4:**
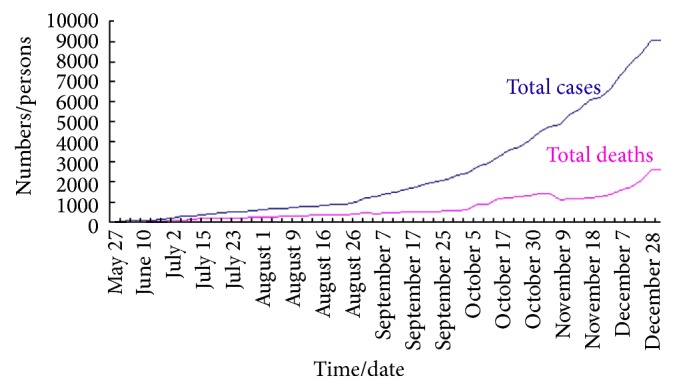
Total cases and deaths in Sierra Leone.

**Figure 5:**
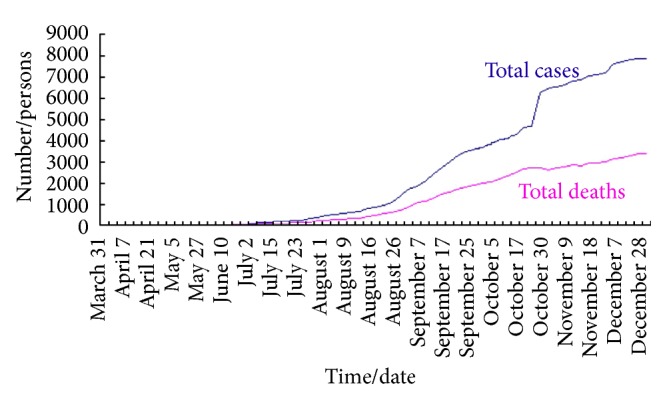
Total cases and deaths in Liberia.

Based on the data in Table 1, we can plot the different graphs which describe the total cases in Sierra Leone, Liberia, and Guinea as shown in Figure 2. Moreover, the graphs in Figures 3 [Fig fig4]–5 reflect the total cases and deaths in those three countries. It is obvious that both numbers have increased rapidly since August.

#### 2.2.2. Model Preparation

**Figure 6:**
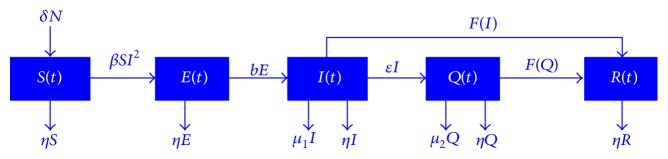
Overview of improved epidemic model.

To fight against Ebola, infectious individuals tend to be isolated to control the spread of the disease, so as to form a separate group known as isolators. We introduce isolators *Q*(*t*) to the model, expand the previous model to SEIQR model, and consider the birth rate *δ* and natural death rate *η*. Let *μ*_1_ and *μ*_2_ represent diseased death rate of infectious patients and isolators, respectively, let *b* represent the proportion of transformation from *E*(*t*) to *I*(*t*), and let *ε* represent the proportion of transformation from *I*(*t*) to *Q*(*t*). Combined with *βSI* ^2^ and maximum treatment capacity, the modifying process is shown in Figure 6.

Also build simultaneous differential equations:

(4) S′t=δN−βSI2−ηS,E′t=βSI2−b+ηE,I′t=bE−μ1+ε+ηI−FI,Q′t=εI−μ2+ηQ−FQ,R′t=FI+FQ−ηR,

where

(5) FI=kI,0≤I≤I0,kI0,I>I0,FQ=kQ,0≤Q≤I0,kQ0,Q>Q0.

We can find that *S*(*t*) + *E*(*t*) + *Q*(*t*) + *I*(*t*) + *R*(*t*) = *N*(*t*) and *N*′(*t*) = (*ε* − *η*)*N* − *μ*_1_ *I* − *μ*_2_ *Q*.

#### 2.2.3. Model Solution and Analysis

Motivated by the works of Du and Xu [8] and Sun and Ma [9] and the discussions above, we simplify the model by the equivalent system of equations with (4) as follows:

(6) S′t=δN−βSI2−ηS,E′t=βSI2−b+ηE,I′t=bE−μ1+ε+ηI−FI.

Then system (6) has a positive invariant set Π = {(*S*, *E*, *I*) ∈ *R*_+_^3^∣0 ≤ *S* + *E* + *I* ≤ *δN*/*η*}.

We obtain the expression of *R*_0_:

(7) $$\begin{array}{c} R_{0}=\frac{b\beta \delta N}{\eta \left(\eta +b\right)\left(\eta +\mu_{1}+\varepsilon +k\right)}. \end{array}$$

If *R*_0_ < 1, then epidemic gets controlled and system (6) has a disease-free equilibrium *E*_0_. If *R*_0_ > 1, then epidemic continues to spread and system (6) has an endemic equilibrium *E* ^*∗*^.

Therefore, decreasing the basic reproduction number is one of the effective ways to eradicate Ebola or control the development of epidemic [10–12]. We can do it from the following aspects:

(i) Increase the value of *k*: the speed of drugs production and distribution will affect the number of people being cured. Speeding up the drug production as well as distributing systemically is a powerful control method.
(ii) Decrease the value of *β*: in this condition, the basic reproduction number will be reduced correspondingly. We will reduce the chances that the Ebola virus carriers contact the susceptible person.
(iii) Increase the value of *ε*: we can insulate the Ebola virus carriers from other susceptible persons.

## 3. How to Distribute Drugs Faster and More Reasonable

### 3.1. The Optimal Route Model and M-S Transportation Assignment Model

This model is to establish how to distribute drugs quickly and reasonably. The aim of the first day is acquiring initial data of each infected district, according to the number of the infected people of each district to distribute drugs [13–15] and the number of susceptible people assigned vaccine. On each of the following days, we need to predict the number of changes to the distribution of drugs, according to the model for the infected and susceptible cases [16–19].

**Figure 7:**
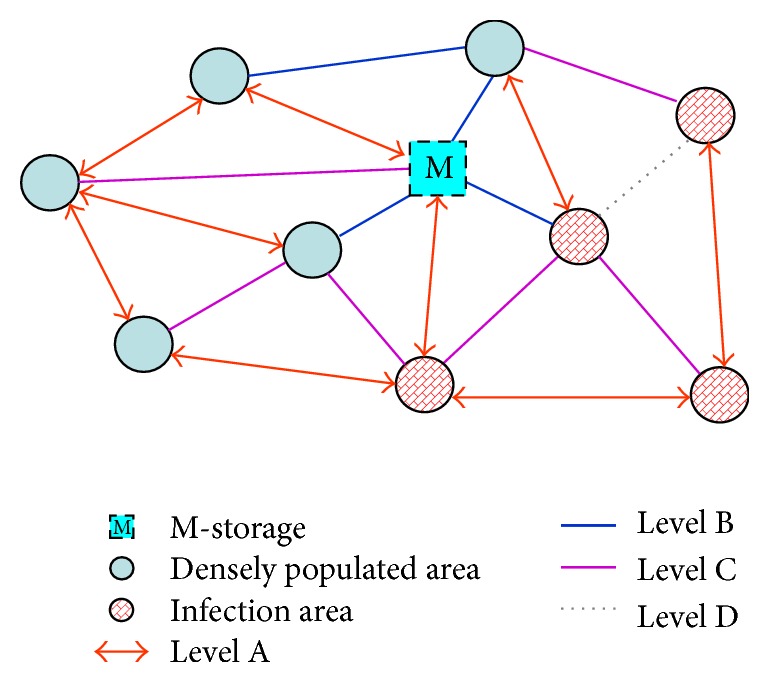
The pictorial diagram of the model.

The pictorial diagram of the model is given in Figure 7.

*(i) The Optimal Route Model*. We design the transport speed without resistance as *v*. Here the relationships between the velocity combined with the road level factors and the original ones are

(8) v~i=μiv,where  i=A,B,C,D,E,μA=1,μB=0.8,μC=0.5,μD=0.1,μE=0.000001.

First, we use two matrixes which contain the information of M-S roads' and each of the area roads' level data:

(9) $$\begin{array}{c} U\left(\;M-S⟶m_{i}\;\right),\\ U^{\prime }\left(\;m_{i}⟶m_{j}\;\right),\\ \;U=\left(\begin{array}{c} \mu^{1}\\ \mu^{2}\\ \vdots \\ \mu^{n} \end{array}\right),\;\;U^{\prime }=\left(\begin{array}{cccc} \mu^{11} & \mu^{12} & \cdots & \mu^{1n}\\ \mu^{21} & \mu^{22} & \cdots & \mu^{2n}\\ \vdots & \vdots & & \vdots \\ \mu^{n1} & \mu^{n2} & \cdots & \mu^{nn} \end{array}\right). \end{array}$$

The velocity combined with the road level factors can be calculated:

(10) V~M−S⟶mi,V~′mi⟶mj,V~=U·v=μ1vμ2v⋮μnv,  V~′=U′·v=μ11vμ12v⋯μ1nvμ21vμ22v⋯μ2nv⋮⋮⋮μn1vμn2v⋯μnnv.

We can also get the distance between the M-S and each area:

(11) $$\begin{array}{c} D\left(\;M-S⟶m_{i}\;\right),\\ L\left(\;m_{i}⟶m_{j}\;\right),\\ \;D=\left(\begin{array}{c} d_{1}\\ d_{2}\\ \vdots \\ d_{n} \end{array}\right),\;\;L=\left(\begin{array}{cccc} l_{11} & l_{12} & \cdots & l_{1n}\\ l_{21} & l_{22} & \cdots & l_{2n}\\ \vdots & \vdots & & \vdots \\ l_{n1} & l_{n2} & \cdots & l_{nn} \end{array}\right). \end{array}$$

After that, we can obtain shortest time data of each routine. Assume that *A* ⊗ *B* means two matrix elements' phase in the corresponding phase, where

(12) T=D⊗V~=d1μ1vd2μ2v⋮dnμnv,T′=L⊗V~′=l11μ11vl12μ12v⋯l1nμ1nvl21μ21vl22μ22v⋯l2nμ2nv⋮⋮⋮ln1μn1vln2μn2v⋯lnnμnnv.

Then we figure out the best routine by using dynamic programming. We design the transport time between area *k* and area *i* (destination) as *t*_*ki*_′, where *k* = 1,2,…, *n*, 1 ≤ *x*_*j*_ ≤ *n*, *x*_*j*_ ≠ *k*, *x*_*j*_ ≠ *i*.

When the routine contains one area road,

(13) $$\begin{array}{c} t_{ki}^{\prime \left(1\right)}=T^{\prime }\left(\;i,k\;\right). \end{array}$$

When the routine contains two area roads,

(14) $$\begin{array}{c} t_{ki}^{\prime \left(2\right)}=T^{\prime }\left(\;i,x_{1}\;\right)+T^{\prime }\left(\;x_{1},k\;\right), \end{array}$$

and so on.

When the routine contains zero area roads,

(15) $$\begin{array}{c} t_{ki}^{\prime \left(n-1\right)}=T^{\prime }\left(\;i,x_{1}\;\right)+\cdots +T^{\prime }\left(\;x_{n-1},k\;\right). \end{array}$$

Next we get the shortest transport time between arbitrary area and destination:

(16) $$\begin{array}{c} t_{k}^{\prime }=\left\{\begin{array}{cc} 0, & k=i,\\ \min\left\{t_{ki}^{\prime \left(1\right)},t_{ki}^{\prime \left(2\right)},\ldots ,t_{ki}^{\prime \left(n-1\right)}\right\}, & k\neq i. \end{array}\right. \end{array}$$

Finally, we can use Dijkstra Matrix Algorithm to get the shortest time on the routine between every two areas:

(17) Dg=t11′t12′⋯t1n′t21′t22′⋯t2n′⋮⋮⋮tn1′tn2′⋯tnn′,where  g=lg⁡n−1lg⁡2.

The shortest time between M-S and every destination can be figured out: *t*_*i*_ = min⁡(*T*(*k*) + *t*_*k*_′). Of course the best routine can also be listed: *H* → *m*_*k*_ → ⋯→*m*_*i*_.

The parameter *m*_*i*_ denotes the population of each area, *l*_*ij*_ denotes the distance between each area, *M* denotes the drug transport terminal, and *d*_*i*_ denotes the distance between terminal and each area.

*(ii) M-S Transportation Assignment Model*. The virus will infect all the time; every day only deliver a certain amount of drug in order to guarantee the timeliness of delivery. Given that the traffic is limited, we can only dispatch the drug from M-S once a day. Hence the distribution of drugs and vaccines should be allotted according to the infection number and arrival time [20–22].

According to the model preparation, we can easily know that

(18) αit=Iit+tiIt+ti·γ1,βit=Sit+tiSt+ti·γ2,θ1t=θ10+λ1η1·t24+1−σ1γ1·t24+1,θ2t=θ20+λ2η1·t24+1−σ2γ1·t24+1,γ1γ2=It+Qtϕ·St·ε,γ1+γ2≤γ,

where 0 ≤ *λ*_1_, *λ*_2_, *σ*_1_, *σ*_2_ ≤ 1, *i* = 1,2,…, *n*.

**Figure 8:**
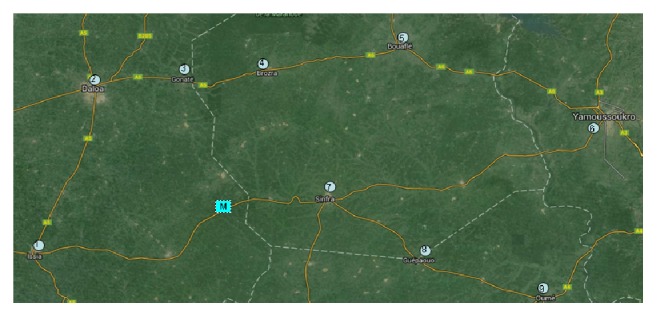
An area of Ivory Coast.

According to our previously established model, we select the Ivory Coast in an area (9 in densely populated areas, a drug storage station) for data simulation. Limited by lack of data search, part of the data (population, prevalence, drug and vaccine production, and storage efficiency) in accordance with the reality of the situation is assumed; see figure position distribution and geographic condition (Figure 8 winning note M in place of M-S).

According to the map information, we use the transport model to calculate the data. We can get the shortest time by the M-S transport of drugs and vaccines to 9 of this densely populated area with Table 2. The basic transport rate is *v* = 60 km/h.

The most efficient routine is in Table 3.

Based on the data in each region, the initial drug inventory is 5000 units. In this inventory, 2000 units are available every day and 3000 units are used to make vaccines. Besides, 4000 units can be transported when the virus infection coefficient *φ* = 0.8, and *ε* = 1 × 10^6^. Because of insufficient data, the above data and population data and the number of patients are assumed. Pharmaceutical distribution plans of each area can be seen in Tables 4 and 5.

From the simulation results, we spend a total of 42 days on complete control of the epidemic. The number of infections in the region is not on the increase. Then the supplies of vaccine and drug need to be sustained.

The parameter *α*_*i*_(*t*) is the amount of drugs distribution in each region, *β*_*i*_(*t*) is the amount of vaccine distribution in each region, *γ* is the massive daily freight, *φ* is the spread of the virus, *ε* is the equilibrium coefficient, *γ*_1_ is the daily traffic volume of drugs, and *γ*_2_ is the daily traffic volume of vaccine.

### 3.2. Medicine Storage and Transport Model

In this section, we will find what kind of storage solution can make the minimum total cost. Given the drug in the corresponding point of storage, we set up the storage model, then we integrate transport vaccine and drugs production costs, and we can get the minimum total cost solution.

#### 3.2.1. Model Preparation

**Figure 9:**
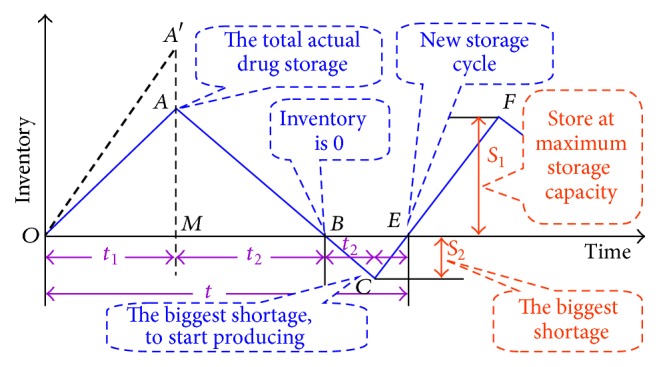
Storage capacity of the model with allowed shortages.

Combining with the general economic ordering quantity model, we should not only consider the relationship between the transport from the pharmaceutical production department to storage and the affected areas needing drugs but also allow the inventory shortage situation. The rate of transport of drugs is *P*, and the rate of drug demand in the affected area is *D* (*P* > *D*). Production sector starts to deliver drugs to storage starting from 0. Then at time *t*_1_, the actual rate of *P* and *D* is increasing; after that, the demand reaches the maximum shortage. After the maximum shortage, we restore supply from that point to supplement shortage and start a new cycle for the drug store.  Figure 9 shows a schematic view of the corresponding period [23–27].

#### 3.2.2. Model Establishing and Solving

We suppose that a cycle length of time for storage is *t*_1_ + *t*_2_ + *t*_3_ + *t*_4_ and use OC, CC, and SC to express preparation cost, storage cost, and shortage cost in a storage cycle, respectively. TC represents the average total cost per unit of time.

By analysis, OC, CC, and SC can be given as

(19) OC=CD,CC=12S1CPt1+t2,SC=12S2CSt3+t4.

So the average total cost per unit of time can be obtained as

(20) TCOC+CC+SCt1+t2+t3+t4=CD+0.5·S1CPt1+t2+0.5·S2CSt3+t4t1+t2+t3+t4.

From Figure 9, we can get

(21) S1P−Dt1=Dt2⟶t1DP−Dt2,t1+t2PP−Dt2,S2P−Dt4=Dt3⟶t4DP−Dt3,t3+t4PP−Dt3,t1+t2+t3+t4PP−Dt2+t3,QDt1+t2+t3+t4=PDP−Dt2+t3.

Restoring data, each length of time can be given as

(22) t3∗=2CDCS1−D/PDCSCP+CS,t2∗=2CDCS1−D/PDCPCP+CS,Q∗=2CDCP+CSDCPCS1−D/P,S1∗=Dt2∗=2DCDCS1−D/PCPCP+CS,S2∗=Dt3∗=2DCDCS1−D/PCSCP+CS.

With generation into the TC we can get the minimum cost:

(23) $$\begin{array}{c} {TC}^{*}=\sqrt{\frac{2DC_{D}C_{S}C_{P}\left(1-D/P\right)}{C_{P}+C_{S}}}, \end{array}$$

where *C*_*D*_ denotes the cost of preparations before transporting drugs, *C*_*P*_ means storage fee of unit drugs in unit time, and *C*_*S*_ means economic losses caused by drug shortages in unit time.

After obtaining the sum cost of the average fee, storage fee, and loss fee in the unit of time, the final total cost can be obtained after adding the corresponding medicine manufacturing cost and transportation cost.

## 4. Conclusions

In summary, we can enlarge the drugs and vaccine supply to control the epidemic. As we can see, the index of *γ* is over one, which means increasing *A* is more efficient in decreasing the virus spread velocity [28]. In addition, when the epidemic was controlled, focusing on the medicine research can be better than the expansion of production on drugs and vaccine. If we can provide more helpful things to people, we do believe that Ebola will be erased in this world [29–33]. By the way, reducing the cost in producing as well as putting more money on the researching is a sustainable plan to face the future that the viruses will become variants.

## Figures and Tables

**Table 1:** The number^*∗*^ of population and total cases and deaths in three countries.

Area	Guinea			Liberia			Sierra Leone		
Month	Population	Total cases	Total deaths	Population	Total cases	Total deaths	Population	Total cases	Total deaths
May 27, 2014	11.2 million	281	186	4.3 million	12	9	6.1 million	16	5
June 24, 2014	11.2 million	390	270	4.3 million	51	34	6.1 million	158	34
July 27, 2014	11.2 million	460	339	4.3 million	329	156	6.1 million	533	233
August 26, 2014	11.2 million	648	430	4.3 million	1378	694	6.1 million	1026	422
September 25, 2014	11.2 million	1103	668	4.3 million	3564	1922	6.1 million	2120	561
October 24, 2014	11.2 million	1598	981	4.3 million	6253	2704	6.1 million	4017	1341
November 23, 2014	11.2 million	2134	1260	4.3 million	7168	3016	6.1 million	6599	1398
December 28, 2014	11.2 million	2597	1607	4.3 million	7862	3384	6.1 million	9004	2582

^*∗*^The date in this table is derived from http://www.who.int/mediacentre/news/ebola/23-october-2014/en/.

**Table 2:** The shortest time of the transport line.

Destination	1	2	3	4	5	6	7	8	9

Hours	1.04375	0.985417	1.516667	3.650417	3.750417	2.770833	1.754167	2.34375	3.079167

**Table 3:** The most efficient routine.

Destination	1	2	3	4	5	6	7
Routine	*M* → 1	*M* → 2	*M* → 3	*M* → 1 → 2 → 3	*M* → 1 → 2 → 3 → 4 → 5	*M* → 6 *M* → 7	*M* → 7 → 8 *M* → 7 → 8 → 9

**Table 4:** Transport schedule on the first day.

City	Population	Patients	Drugs	Vaccine
1	511154	112	41	471
2	12211	11	5	14
3	121233	0	0	132
4	56454	0	0	55
5	64441	1	1	63
6	2023756	2133	767	1930
7	68782	12	65	161
8	124144	0	0	291
9	468444	0	0	457

**Table 5:** Transport schedule when the epidemic is controlled.

City	Population	Patients	Drugs	Vaccine
1	511154	131	48	495
2	12211	11	5	14
3	121233	0	0	139
4	56454	0	0	68
5	64441	0	0	79
6	2023756	1683	662	2513
7	68782	4	663	348
8	124144	0	0	700
9	468444	0	0	3337
